# Lithological and granulometric data for the upper sedimentary layer of the Chaun Bay, East Siberian Sea

**DOI:** 10.1016/j.dib.2022.108813

**Published:** 2022-12-09

**Authors:** Alexander S. Ulyantsev, Elena A. Streltsova, Alexander N. Charkin

**Affiliations:** a Shirshov Institute of Oceanology, Russian Academy of Sciences, Moscow 117997, Russia; b Il'ichev Pacific Oceanological Institute, Far Eastern Branch of the Russian Academy of Sciences, Vladivostok 690041, Russia

**Keywords:** Arctic shelf, Bottom sediments, Lithology, Sorting coefficient, Standard deviation, Skewness, Kurtosis

## Abstract

The article provides a data on 160 samples of bottom sediments obtained from 48 stations during 60th cruise of R/V Akademik Oparin in the East Siberian Sea in Autumn 2020 (26 September – 11 November). It contains mean diameter of the particles, sorting coefficient, standard deviation, skewness, and kurtosis, values of percentiles (p5, p10, p16, p25, p50, p75, p84, p90, p95), lithology and mass percentage of >2 mm, 1-2 mm, 0.5-1 mm, 250-500 µm, 125-250 µm, 63-125 µm, 31-63 µm, 10-31 µm, 2-10 µm, and <2 µm fractions. The bottom sediments have been sampled in Chaun Bay of the East Siberian Sea from 9 to 21 October 2020 with Ekman (0.25 m^2^) and Van Veen (0.05 m^2^) samplers. The grain size data was obtained from laser diffraction method using a SHIMADZU SALD 2300 particle analyser. The data provides an overview on lithology and grain size properties of bottom sediments that will be useful to understand risks for climate changes in the Arctic. The data will help the researchers who work on the Arctic to assess a relationship between grain size properties of the bottom sediments and it sorption potential. A principal component analysis (PCA) can be performed to identify key parameters of the bottom sediments in assessment of sedimentation rates and it changes in the Arctic. Additionally, the data can be used in mapping a spatial distribution of above mentioned parameters for better understanding sedimentation rate changes in the East Siberian Sea and adjacent seas as well.

**Specifications Table**

**Table:** 

Subject	Earth and Planetary Sciences
Specific subject area	Bottom sediments, lithology, grain size
Type of data	Tables
How data were acquired	The samples of bottom sediments were collected in the Chaun Bay during 60th cruise of R/V Akademik Oparin to the Arctic seas in the Autumn 2020 (26 September – 11 November). Data was obtained after a sieving procedure from laser diffraction method using a SHIMADZU SALD 2300 particle analyser.
Data format	Raw and analysed data (recalculated from size spectra)
Description of data collection	Data were obtained from analysis of the bottom sediments sampled with Ekman (0.25 m^2^) and Van Veen (0.05 m^2^) grab samplers. Analytical procedures include a sieving and laser diffraction measurements.
Data source location	The samples of were collected in the Arctic Ocean, East Siberian Sea, Chaun Bay. GPS coordinates of sampling stations are provided in Table 2. Measurements were performed at the Laboratory of the ocean chemistry, Shirshov Institute of Oceanology, Russian Academy of Sciences, Moscow, Russia.
Data accessibility	Repository name: Mendeley Data identification number: 10.17632/r9wm2myzkv.2 [1] Direct URL to data: https://data.mendeley.com/datasets/r9wm2myzkv/2
Related research article	A.S. Ulyantsev, A.N. Charkin, V.L. Syomin, L.A. Kireenko, S.A. Botsul, A.E. Leusov, P.Y. Semkin, S.P. Kukla, Geological Studies of the Upper Sedimentary Strata of Chaun Bay during Cruise 60 of the R/V Akademik Oparin, Oceanology. 61 (2021) 584–585. 10.1134/S0001437021040159.

## Value of the data

• The data provides detailed overview on lithology and grain size properties of bottom sediments from Chaun Bay that will be useful to understand risks for climate changes in the Arctic.
• The data will help the researchers who work on the Arctic to assess a relationship between grain size properties of the bottom sediments and it sorption potential.
• The data will be useful as a baseline for potential contaminant level assessment in Chaun Bay.
• The data can be used in mapping spatial distribution of mean diameter of the particles, sorting coefficient, standard deviation, skewness, and kurtosis for better understanding a sedimentation rate changes in the East Siberian Sea and adjacent seas as well.

## 1 Objective

The data represents a strong set that can help to characterise lithodynamics and sedimentation rates in the East Siberian Sea as a part of periglacial Arctic shelf. It is useful in assessment of the environmental state and potential risks of climate changing in the Arctic basin. A principal component analysis (PCA) can be performed to identify key parameters of the bottom sediments in assessment of sedimentation rates and it changes in the Arctic.

## 2 Data Description

The samples of bottom sediments were obtained during 60^th^ cruise of R/V Akademik Oparin to the East Siberian Sea in Autumn 2020 (26 September – 11 November). The expedition was organised by Il'ichev Pacific Oceanological Institute FEB RAS (Vladivostok) in collaboration with Lomonosov Moscow State University (Moscow) and Shirshov Institute of Oceanology RAS (Moscow). The bottom sediments have been sampled within Chaun bay from 9 to 21 October 2020 according to IAEA-TECDOC-1360 protocol [2] using Ekman (0.25 m^2^) and Van Veen (0.05 m^2^) grab samplers. The sampling procedures have been performed during the absence of land-fast and sea ice. Table 1 contains a list of the calculated grain size parameters of the bottom sediments. Table 2 contains station numbers, GPS coordinates, sea water depth, sampling dates, and lithological characteristics of the sampled bottom sediments.

**Table 1:** List of the calculated grain size parameters. p — percentiles of the particle size distribution of each sample calculated in mm; ϕ — percentiles of the particle size distribution of each sample calculated according to [3].

Parameter	Equation	Refs.
Mean diameter	$M_{Z}=\frac{p5+p16+p50+p84+p95}{5}$	[4]
Sorting coefficient	$S_{o}=\sqrt{\frac{p75}{p25}}$	[4]
Standard deviation	$\sigma_{I}=\frac{\phi 84-\phi 16}{4}+\frac{\phi 95-\phi 5}{6.6}$	[5]
Skewness	$Sk_{I}=\frac{\phi 16+\phi 84-2\phi 50}{2(\phi 84-\phi 16)}+\frac{\phi 5+\phi 95-2\phi 50}{2(\phi 95-\phi 5)}$	[5]
Kurtosis	$K_{G}=\frac{\phi 95-\phi 5}{2.44(\phi 75-\phi 25)}$	[5]

**Table 2:** Station numbers, GPS coordinates, sea water depth and lithological characteristics of the sampled bottom sediments.

Station	Latitude, °N	Longitude, °E	Water depth, m	Horizon, cm	Sampling date	Lithology	Colour
03	69.772	170.503	14	0-2	09.10.2020	Silty-clayey mud with sand	Light brown
				2-5	09.10.2020	Silty-clayey mud with sand	Olive
				5-10	09.10.2020	Silty clay, hydrotroilite lenses	Dark grey
				10-20	09.10.2020	Silty clay, hydrotroilite lenses	Dark grey
04	69.759	170.266	11	0-2	09.10.2020	Clayey mud	Light brown
				2-5	09.10.2020	Clayey mud	Olive
				5-10	09.10.2020	Clay, hydrotroilite lenses	Dark grey
				10-20	09.10.2020	Clay, hydrotroilite lenses	Dark grey
05	69.732	170.274	21	0-2	09.10.2020	Silty-clayey mud with sand	Brown
				2-5	09.10.2020	Silty-clayey mud	Olive
				5-10	09.10.2020	Silty clay, hydrotroilite inclusions	Dark grey
				10-20	09.10.2020	Silty clay, hydrotroilite inclusions	Dark grey
06	69.720	170.288	22	0-2	09.10.2020	Silty-clayey mud with sand	Light brown
				2-5	09.10.2020	Silty-clayey mud	Olive
				5-10	09.10.2020	Silty clay, hydrotroilite inclusions	Grey
				10-20	09.10.2020	Silty clay, hydrotroilite inclusions	Grey
07	69.761	169.728	25	0-2	10.10.2020	Silty-clayey mud	Light brown
				2-5	10.10.2020	Silty-clayey mud	Olive
				5-10	10.10.2020	Silty-clayey mud	Light grey
08	69.578	170.122	14	0-2	11.10.2020	Silty-clayey mud	Brown
				2-5	11.10.2020	Silty-clayey mud	Olive
				5-10	11.10.2020	Silty clay, hydrotroilite lenses	Grey
09	69.553	170.062	15	0-2	11.10.2020	Clayey mud	Brown
				2-5	11.10.2020	Clayey mud	Olive
				5-10	11.10.2020	Clay, hydrotroilite inclusions	Grey
10	69.541	169.972	16	0-2	11.10.2020	Clayey mud	Brown
				2-5	11.10.2020	Clayey mud	Olive
				5-10	11.10.2020	Clay	Grey
31	69.509	170.390	12	0-2	12.10.2020	Silty-clayey mud	Brown
				2-5	12.10.2020	Silty-clayey mud	Olive
				5-10	12.10.2020	Silty-clayey mud	Grey
				10-20	12.10.2020	Silty clay	Dark grey
32	69.349	170.549	11	0-2	12.10.2020	Silty-clayey mud	Dark Brown
				2-5	12.10.2020	Silty-clayey mud	Grey
				5-10	12.10.2020	Silty clay, hydrotroilite lenses	Dark grey
33	69.358	170.146	16	0-2	12.10.2020	Silty-clayey mud	Brown
				2-5	12.10.2020	Silty-clayey mud	Olive
				5-10	12.10.2020	Silty clay, hydrotroilite lenses	Grey
				10-20	12.10.2020	Silty clay, hydrotroilite lenses	Grey
34	69.554	169.695	20	0-2	12.10.2020	Clayey mud	Brown
				2-5	12.10.2020	Clayey mud	Olive
				5-10	12.10.2020	Clayey mud	Dark olive
				10-20	12.10.2020	Clayey mud, hydrotroilite inclusions	Grey
42	69.640	170.098	17	0-2	13.10.2020	Silty-clayey mud with sand	Brown
				2-5	13.10.2020	Silty-clayey mud	Olive
				5-10	13.10.2020	Silty clay	Light grey
				10-20	13.10.2020	Silty clay	Grey
43	69.637	170.112	19	0-2	13.10.2020	Silty-clayey mud with sand	Brown
				2-5	13.10.2020	Silty-clayey mud with sand	Olive
				5-10	13.10.2020	Silty clay with sand	Grey
				10-20	13.10.2020	Silty clay, hydrotroilite inclusions	Dark grey
44	69.632	170.132	18	0-2	13.10.2020	Silty-clayey mud	Brown
				2-5	13.10.2020	Silty-clayey mud	Olive
				5-10	13.10.2020	Silty clay, hydrotroilite lenses	Dark grey
				10-20	13.10.2020	Silty clay, hydrotroilite lenses	Dark grey
57	69.267	169.772	16	0-2	14.10.2020	Silty mud	Light brown
				2-5	14.10.2020	Silty-clayey mud	Light grey
				5-10	14.10.2020	Clay	Grey
				10-20	14.10.2020	Clay, hydrotroilite inclusions	Dark grey
58	69.182	169.864	15	0-5	14.10.2020	Silty-sandy-clayey mud	Olive
				5-10	14.10.2020	Silty-sandy clay, hydrotroilite inclusions	Dark grey
				10-20	14.10.2020	Silty-sandy clay, hydrotroilite lenses	Dark grey
59	69.209	170.195	16	0-2	14.10.2020	Silty-clayey mud with sand	Brown
				2-5	14.10.2020	Silty-clayey mud with sand	Olive
				5-10	14.10.2020	Silty clay	Grey
				10-20	14.10.2020	Silty clay	Dark grey
60	69.201	170.569	12	0-2	14.10.2020	Silty-clayey mud with sand	Brown
				2-5	14.10.2020	Silty-clayey mud with sand	Olive
				5-10	14.10.2020	Silty clay	Dark grey
				10-20	14.10.2020	Silty clay	Dark grey
61	69.372	169.744	18	0-1	15.10.2020	Clayey mud	Light brown
				1-5	15.10.2020	Clayey mud	Olive
				5-10	15.10.2020	Clayey mud, hydrotroilite inclusions	Grey
				10-20	15.10.2020	Clayey mud, hydrotroilite inclusions	Grey
62	69.053	170.380	13	0-2	15.10.2020	Silty-clayey mud with sand	Dark brown
				2-5	15.10.2020	Silty-clayey mud with sand	Olive
				5-10	15.10.2020	Silty-clayey mud with sand	Grey
				10-20	15.10.2020	Silty clay	Dark grey
63	68.967	170.302	13	0-2	15.10.2020	Sand	Red-brown
				2-5	15.10.2020	Sand	Light grey
				5-10	15.10.2020	Silty sand, hydrotroilite inclusions	Grey
				10-20	15.10.2020	Silty sand, hydrotroilite inclusions	Dark grey
64	68.879	169.978	11	0-2	15.10.2020	Sand	Red-brown
				2-10	15.10.2020	Sand	Olive
65	68.888	169.728	10	0-2	15.10.2020	Sand	Brown
				2-10	15.10.2020	Sand	Olive
66	69.052	169.974	15	0-2	16.10.2020	Silty-clayey mud with sand	Brown
				2-5	16.10.2020	Silty-clayey mud with sand	Olive
				5-10	16.10.2020	Silty clay, hydrotroilite inclusions	Grey
				10-20	16.10.2020	Silty clay, hydrotroilite inclusions	Dark grey
67	69.043	169.726	14	0-2	16.10.2020	Silty mud	Brown
				2-5	16.10.2020	Sandy-silty mud, shale inclusions	Olive
				5-10	16.10.2020	Silty sand	Grey
				10-20	16.10.2020	Silty sand	Grey
68	69.075	169.419	12	0-5	16.10.2020	Sandy-silty mud	Olive
				5-10	16.10.2020	Sandy silt	Grey
69	69.082	169.460	12	0-2	16.10.2020	Sandy mud	Light brown
				2-5	16.10.2020	Sandy mud	Brown
				5-10	16.10.2020	Sandy silt, hydrotroilite inclusions	Dark grey
				10-20	16.10.2020	Sandy silt	Dark grey
70	69.134	169.335	11	0-3	16.10.2020	Sand	Brown
				3-10	16.10.2020	Sand with clay lenses	Olive
71	69.218	169.051	10	0-5	17.10.2020	Sandy mud	Dark brown
				5-10	17.10.2020	Sandy mud, hydrotroilite lenses	Dark grey
72	69.369	169.362	10	0-5	17.10.2020	Sand	Olive
				5-10	17.10.2020	Sandy mud	Grey
				10-20	17.10.2020	Silty clay with sand, hydrotroilite saturation	Dark grey
73	69.558	169.523	12	0-5	17.10.2020	Sandy mud	Olive
				5-10	17.10.2020	Sandy mud	Grey
74	69.676	169.480	11	0-2	17.10.2020	Sandy mud	Olive
				2-5	17.10.2020	Sandy mud	Grey
				5-10	17.10.2020	Sandy mud	Dark grey
75	68.832	170.372	6	0-10	18.10.2020	Sandy mud	Dark grey
76	68.871	170.228	10	0-3	18.10.2020	Sand	Red-brown
				3-5	18.10.2020	Sandy mud	Grey
				5-10	18.10.2020	Sandy mud, hydrotroilite lenses	Dark grey
77	68.958	170.358	11	0-2	18.10.2020	Sandy mud	Red-brown
				2-5	18.10.2020	Sandy mud	Olive
				5-10	18.10.2020	Silty sand	Grey
				10-20	18.10.2020	Silty sand, hydrotroilite inclusions	Dark grey
78	69.064	169.420	11	0-2	19.10.2020	Sandy-silty mud	Olive
				2-5	19.10.2020	Sandy-silty mud	Light grey
				5-10	19.10.2020	Sandy silt	Grey
				10-20	19.10.2020	Sandy silt	Dark grey
79	69.040	169.459	10	0-2	19.10.2020	Sandy-silty mud	Olive
				2-5	19.10.2020	Sandy-silty mud	Light grey
				5-10	19.10.2020	Sandy silt	Grey
				10-20	19.10.2020	Sandy silt, hydrotroilite lenses	Dark grey
80	69.008	169.502	9	0-2	19.10.2020	Sandy-silty mud	Olive
				2-5	19.10.2020	Sandy-silty mud, shale inclusions	Light grey
				5-10	19.10.2020	Sandy silt, shale inclusions	Grey
81	69.069	169.382	5	0-10	19.10.2020	Sandy mud	Grey
82	69.065	169.359	2	0-10	19.10.2020	Sand	Dark grey
86	70.064	170.497	22	0-2	20.10.2020	Silty-clayey mud	Olive
				2-5	20.10.2020	Clayey mud	Light grey
				5-10	20.10.2020	Clayey mud	Grey
				10-20	20.10.2020	Clayey mud, hydrotroilite lenses	Dark grey
88	70.017	170.020	16	0-2	20.10.2020	Silty mud	Olive
				2-5	20.10.2020	Silty mud	Light grey
				5-10	20.10.2020	Silty mud	Grey
				10-20	20.10.2020	Silty mud, hydrotroilite inclusions	Grey
90	69.961	169.714	14	0-2	20.10.2020	Silty-clayey mud with sand	Olive
				2-5	20.10.2020	Silty-clayey mud with sand	Light grey
				5-10	20.10.2020	Clayey silt with sand lenses	Grey
				10-20	20.10.2020	Clayey silt with sand lenses	Grey
94	70.168	168.878	18	0-5	21.10.2020	Silty-clayey mud with sand	Olive
				5-10	21.10.2020	Silty clay, hydrotroilite inclusions	Dark grey
				10-20	21.10.2020	Silty clay, hydrotroilite inclusions	Dark grey
95	70.145	169.807	20	0-2	21.10.2020	Silty-clayey mud with sand	Olive
				2-5	21.10.2020	Silty-clayey mud with sand	Light grey
				5-10	21.10.2020	Silty clay, hydrotroilite inclusions	Grey
				10-20	21.10.2020	Silty clay, hydrotroilite inclusions	Dark grey
97	70.447	170.076	29	0-2	21.10.2020	Clayey mud	Olive
				2-5	21.10.2020	Silty-clayey mud with sand	Light grey
				5-10	21.10.2020	Silty-clayey mud with sand, hydrotroilite lenses	Grey
				10-20	21.10.2020	Silty clay, hydrotroilite lenses	Grey
99	70.800	170.432	30	0-2	21.10.2020	Clayey mud	Olive
				2-5	21.10.2020	Silty-clayey mud with sand	Grey
				5-10	21.10.2020	Silty clay, hydrotroilite lenses	Dark grey
				10-20	21.10.2020	Silty clay, hydrotroilite lenses	Dark grey

## 3 Experimental Design, Materials and Methods

The sampling horizon for each station was chosen based on lithology and colour of sediments. All samples for grain size analysis were put into zip-lock polypropylene bags and stored at +8°C for analytical procedures. Totally 160 samples were analysed using a laser diffraction method [6] according to ISO 13320:2020 [7]. Before the analytical procedures all samples were fractionated by wet sieving using 2, 1, 0.5, 0.25, 0.125, and 0.063 mm sieve sizes. After the sieving procedure all fraction were dried and weighted. Fraction < 0.063 mm was analysed by laser diffraction using a SALD 2300 particle analyzer (Shimadzu, Japan). Analytical conditions were 2000 rpm pump speed, 40 W ultrasound sonification power (32 kHz), 2500 Hz scanning frequency, sonification time – 1 min. Milli-Q water was used as a dispersant and a blank. All laser diffraction analyses were done in triplicate and averaged using WingSALD software. The calculated parameters (mean diameter, sorting coefficient, standard deviation, skewness, and kurtosis) and values of percentiles (p5, p10, p16, p25, p50, p75, p84, p90, p95) are given in Supplementary Table 1. The relative mass contributions (in %) of size fractions are also given in Supplementary Table 1 and expressed as follows: >2 mm, 1-2 mm, 0.5-1 mm, 250-500 µm, 125-250 µm, 63-125 µm, 31-63 µm, 10-31 µm, 2-10 µm, and <2 µm.

## Ethics Statements

No human subjects, animal experiments, or data collected from social media platforms were used.

## CRediT authorship contribution statement

**Alexander S. Ulyantsev:** Writing – original draft, Visualization, Project administration, Conceptualization, Funding acquisition. **Elena A. Streltsova:** Formal analysis, Validation, Investigation. **Alexander N. Charkin:** Investigation, Funding acquisition.

## Declaration of Competing Interest

The authors declare that they have no known competing financial interests or personal relationships that could have appeared to influence the work reported in this paper.

## Data Availability

Grain size properties of bottom sediments from the Chaun Bay, East Siberian Sea (Original data) (Mendeley Data)

## Appendix Supplementary materials

Image, application 1
